# S gene mutations of HBV in children with HBV-associated glomerulonephritis

**DOI:** 10.1186/1743-422X-9-59

**Published:** 2012-03-05

**Authors:** Hongzhu Lu, Hui Zhu, Jianhua Zhou

**Affiliations:** 1 Department of Pediatrics, Tongji Hospital, Tongji Medical College, Huazhong University of Science and Technology, Wuhan 430030, People's Republic of China; 2 Department of Pediatrics, the First Affiliated Hospital, Clinical Medical College, Yangtze University, Jingzhou, Hubei, People's Republic of China

## Abstract

**Background:**

Hepatitis B virus-associated glomerulonephritis (HBV-GN) is a kind of immune complex-induced glomerulonephritis. The present study was designed to determine whether mutation of Hepatitis B virus (HBV) S gene is associated with glomerulonephritis in Chinese children.

**Methods:**

Total 53 subjects, including 30 HBV-GN, 5 nephrosis with HBV carriers (control group 1), and 18 HBV carriers (control group 2) were included in this study. Polymerase chain reaction (PCR) was used to detect the HBV-GN S gene mutation.

**Results:**

(1) The serotype of HBV was adw in the majority (52/53) of subjects, and was adr in only 1 subject in the control group 2; (2) the genotype of HBV was the type B in 51 subjects, the type E in 1 HBV-GN child, and the type C in 1 HBV carrier; (3) Seventeen point mutations in the S gene of HBV were identified in 21 of 30 (70%) HBV-GN patients. Among them, 16 of 21 (76.2%) mutations may cause amino acid substitutions of the HBV proteins, which occur predominantly (11/16 mutations) at threonine, serine or tyrosine phosphorylation sites of mitogen-activated protein kinase (MAPK) or protein tyrosine kinase (PTK). (4) In addition, single nucleotide mutations without amino acid substitutions (same sense mutation) were found in 2 subjects in each control group and 5 subjects in HBV-GN group.

**Conclusions:**

HBV S gene mutations and the subsequent amino acid substitutions in HBV proteins were found in most children with HBV-GN, suggesting that these mutations may play an important role in the pathogenesis of HBV-GN.

## Background

Hepatitis B viruses (HBV) are well-recognized as the causes of chronic hepatitis, cirrhosis, and hepatocellular carcinoma. In addition, HBV infection is also associated with a spectrum of extrahepatic manifestations [1]. Hepatitis B is prevalent in China and hepatitis B virus-associated glomerulonephritis (HBV-GN) is one of the common renal damages secondary to HBV infection in Chinese children [2]. The mechanism of HBV-GN is generally believed to be related to HBV-related immune reactions [3]. This is based on the findings that HBV viral antigens were detected in kidney tissue [4,5], and further HBV surface antigen (HBsAg) and HBV nucleus antigen were detected in the glomerular deposits [6]. However, the precise pathogenesis of HBV-GN is not fully understood. The HBsAg was present in all children with HBV-GN [6] and also many HBV carriers. Thus, we hypothesized that HBV S gene mutations and their subsequent amino acid substitutions in HBsAg may alter antigenity of HBsAg in HBV-GN, causing immuno-reactive complex deposited in subepithelial areas in glomeruli. Therefore, in the present study, we used the PCR technique to examine the S gene sequence of HBV in order to determine whether characteristic variants of HBV S gene are associated with HBV-GN in Chinese children.

## Results

### Clinical characteristics of the participants

Thirty patients (29 male, 1 female), at the age of 7.7 ± 2.8, were diagnosed as HBV-GN. Percutaneous renal biopsy was performed in these patients. All patients had proteinuria, 21 children had nephrotic syndrome, and 9 patients had proteinuria and hematuria. Result of liver function test was normal in 21 patients. Serology markers of HBV of the participants were shown in Table 1.

**Table 1:** Serology markers of HBV in children with HBV-GN

Findings	Number of participants
HBsAg positive	30

HBeAg positive	19

HBV DNA positive	30

ALT/AST increased	14

ALT: alanine aminotransferase; AST: aspartate aminotransferase

### HBV serotype

HBV S gene contains 678 bp, which encode 226 amino acids of HBsAg major protein. There are 2 subtype determinants (d/y and w/r) in the major protein. Therefore, HBV has 4 serotypes (adw, adr, ayw and ayr) based upon the 122nd amino acid (Lys/Arg, d/y) and the 160th amino acid (Lys/Arg, w/r) in S protein [7]. We detected 53 subjects, in whom 52 were adw and 1 was adr in the control group 2.

### HBV genotype

HBV was classified into 8 genotypes according to genotype-specific restriction enzyme sites (A, B, C, D, E, F, G, and H).

The HBV genotype distributions in the different groups were shown in Table 2. There was no difference between HBV-GN and control groups. Most of them were the genotype B, only 1 HBV-GN was the genotype E, and 1 HBV carrier was the genotype C (Table 2).

**Table 2:** HBV genotype in HBV-GN and control groups

Group (Number of cases)	B	C	E
HBV-GN (30)	29	0	1

Control 1 (5)	5	0	0

Control 2 (18)	17	1	0

### Mutations in HBV S gene

**Figure 1:**
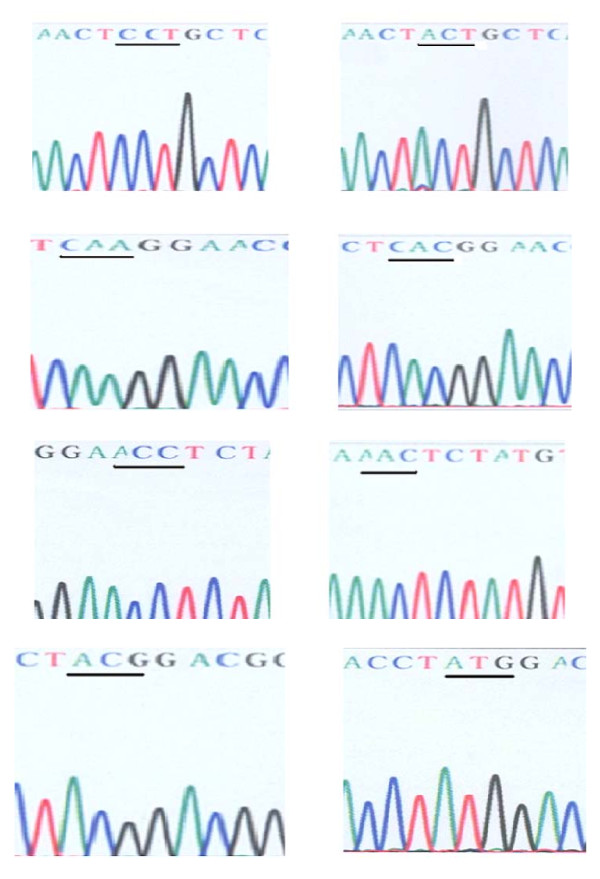
**Point mutation-caused amino acid substitutions in HBV surface antigen (HBsAg) were shown in children with HBV-GN**. Panel A, Pro 127 Thr (CCT→ACT); Panel B, Gln 129 His (CAA→CAC); Panel C, Thr 131 Asn (ACC→AAC); Panel D, Thr 143 Met (ACG→ATG).

The HBV S gene sequences in 53 subjects were compared with AY167097.1, an endemic HBV strain of serotype adw and genotype B in China. The results were shown in Tables 3 and 4. The chromatograph sequences of mutations in some HBV-GN were shown in Figure 1.

**Table 3:** HBV S gene mutations and the inferred amino acid substitutions in children with HBV-GN

**Case No**.	amino acid loci in S region	Codon change	Amino acid substitutions
1	94	TTG**→**TCG	Leu**→**Ser

2	161	TTC**→**TGC	Phe**→**Cys

3	143	TCG**→**ATG	Ser**→**Met
	161	TTC**→**TGC	Phe**→**Cys
	200	TAT**→**TTT	Tyr**→**Cys
	122	AAA**→**AAG	Lys
	125	ACA**→**ACG	Thr
	136	TCA**→**TCC	Ser
	155	TCT **→**TCC	Ser

4	122	AAA**→**AAG	Lys

5	164	GAG**→**GGG	Glu**→**Gly

6	200	TAT**→**TTT	Tyr**→**Cys

7	125	ACA**→**ACG	Thr

8	143	TCG**→**ATG	Ser**→**Met

9	204	AGT**→**AGA	Ser**→**Arg
	125	ACA**→**ACG	Thr

10	96	GTT**→**GGT	Val**→**Gly
	200	TAT**→**TTT	Tyr**→**Cys

11	161	TTC**→**TGC	Phe**→**Cys

12	155	TCT **→**TCC	Ser

13	122	AAA**→**AAG	Lys
	136	TCA**→**TCC	Ser

14	45	ACA**→**ACT	Thr

15	127	CCT**→**ACT	Pro**→**Thr

16	143	TCG**→**ATG	Ser**→**Met
	161	TTC**→**TGC	Phe**→**Cys
	122	AAA**→**AAG	Lys
	125	ACA**→**ACG	Thr
	136	TCA**→**TCC	Ser
	155	TCT **→**TCC	Ser

17	129	CAA**→**CAC	Gln**→**His
	119	GGA**→**GGC	Gly

18	129	CAA**→**CAC	Gln**→**His
	119	GGA**→**GGC	Gly
	125	ACA**→**ACG	Thr

19	129	CAA**→**CAC	Gln**→**His
	125	ACA**→**ACG	Thr

20	175	CAT→CGT	His**→**Arg

21	198	ATG**→**ACG	Met**→**Thr

others	No mutation		

**Table 4:** Gene mutations in HBV S region in control groups

Control groups	**Case No**.	Amino acid loci	Gene mutations	Amino acid
Nephrosis with HBV carrier	1	45	ACA**→**ACT	Thr
	2	118	ACC**→**ACT	Thr
		122	AAA**→**AAG	Lys

HBV carrier	1	54	CAA**→**CAG	Gln
	2	115	ACA**→**ACG	Thr
		190	GTC**→**GTT	Val

Seventeen point mutations were identified in 21 of 30 (70%) HBV-GN patients. Among them, 16 of 21(76.2%) mutations were involved in amino acid substitutions. Moreover, 11/16 (68.8%) were involved amino acid substitutions at Threonine, Serine or Tyrosine phosphorylation sites of mitogen-activated protein kinase (MAPK) and protein tyrosine kinase (PTK) in HBV proteins. In addition, single nucleotide mutations without amino acid substitution (same sense mutation) were found in 2 subjects in each control group and 5 subjects in HBV-GN group.

## Discussion

Recognition of an association between chronic HBV and glomerular disease dates back to the 1970s [8]. This syndrome occurs mainly in children, predominantly in male in HBV endemic areas of the world. The most typical presentations of HBV-GN include nephrotic syndromes and memberanous glomerulonephropathy. In these children, liver function tests are frequently normal. About 60% of patients show spontaneous remission [9]. Although many patients with renal diseases display blood HBsAg-positive, only a few patients have detectable HBV antigens in the kidney. However, HBsAg immune complex deposits are detectable in kidney in some patients with viral hepatitis but without renal diseases [10]. Therefore, the potential role of HBsAg in pathogenesis of HBV-GN remains unclear.

In present study, we attempted to study whether HBV serotype or genotype characteristics are associated with HBV-GN. We found that the HBV serotypes and genotypes were similar in both HBV-GN and control groups. The endemic HBV strain was the serotype adw and the genotype B in south China [11]. Therefore, it was not surprising that all children with HBV-GN and the majority (22 of 23) subjects in control groups were the HBV genotype B. Since the HBV serotype and genotype have not been reported previously in HBV-GN in children, we were not sure whether HBV strain other than the serotype adw and the genotype B might be associated with HBV-GN in children. It is interesting that the serotype ayw and genotype A of HBV are predominant in HBV-GN in South Africa with different endemic HBV strain infection, where the serotype adw and the genotype B were not as epidemic as in China [12-14]. Kim et al. [15] reported 209 patients with HBV infection, in which the adr, adw and ayr serotypes were found in 193, 12 and 1 subjects, respectively. The extraordinary predominance of the genotype C was found in chronic HBV patients in Korean, suggesting that the clinical manifestations of chronic HBV patients in Korean are different from those in other Asian countries.

Although no specific HBV genotype and serotype were identified in HBV-GN, it was interesting that HBV S gene mutations were found in most children with HBV-GN (21 of 30, 70%). Moreover, most of them (16 of 21, 76.2%) were involved in point mutations which may result in amino acid substitutions in HBsAg protein. Threonine, serine and tyrosine are the most likely amino acid substitution sites. Eleven of 16 children with missense mutation in HBV S gene showed lose or gain of these three amino acids. Kim et al. [16] also found similar amino acid substitutions in HBsAg in 6 of 7 children with HBV associated membranous nephropathy (HBV-MN). Furthermore, one of the substitutions in each subject was involved in serine gaining. It is well known that serine and threonine could be phosphorylated by MAPK and tyrosine could be phosphorylated by PTK. The phosphorylation is critical to the intracellular function of S proteins, large proteins and middle proteins. The important amino acid substitutions secondary to their gene mutations may result in different biological behavior in HBV-infected cells. The significance of the present findings is unclear. Since no similar mutations were found in HBV carriers and those with primary nephrosis, we believe that the S gene mutations observed in the present study may play an important role in the pathogenesis of HBV-GN.

The substitution of amino acid in HBV surface protein may change not only the intracellular biological behavior of HBV, but also the immune response of human body. Many amino acid substitutions were identified in the "α" determinant of HBsAg, which was crucial in the binding of neutralizing antibodies. Thus, virus with mutations could escape from clearance by human immune system [17,18]. The persistent infection of HBV is prerequisite for the development of HBV-GN. Kim et al. [16] demonstrated that deletions and point mutations in the HBV pre-S1, pre-S2 region, and point mutations in the HBV S region, especially the "α" determinant region, were frequently discovered in renal tissue of children with HBV-MN. Further, changes in HBV surface protein may facilitate the binding of HBsAg to its receptors in glomerular epithelial cells, a process called "plant of antigen in situ", which may have triggered the development of HBV-MN.

Not all children with HBV-GN showed point mutations in HBV S gene. We did not detect the genome sequence of HBV. It could not be exclude that point mutations existed in other regions of HBV genome. The subepithelial deposition of HBsAg and their antibodies played a key role in the formation of HBV-MN.

## Conclusions

The importance of HBsAg encourages us to explore the role of HBV S gene mutations in HBV-GN. The present data suggest that HBV S gene mutations are closely associated with HBV-GN. Further HBV genome analysis is required to confirm the findings observed in the present study.

## Methods

### Procedures to detect point mutations of HBV S gene by PCR and direct sequencing analysis

Sera samples from child participants → DNA purification → HBV S gene amplification → 1.5% Agarose gel electrophoresis → PCR-DNA purification → Automatic sequencing → Comparing with AY167097.1

### Participants and samples

Thirty children with HBV-GN diagnosed by renal biopsy from Department of Pediatrics, Tongji Hospital, Tongji Medical College, Huazhong University of Science and Technology, from February 1992 to October 2003, were included in this study. Additional 5 nephrotic children with HBV carrier but not HBV-GN served as control group 1, and 18 HBV carriers without nephrosis as control group 2. The blood was collected from these children and the sera were isolated at 4°C and stored at -20°C until PCR assays. Informed consent was obtained from all of the patients recruited into the study, according to the ethical principles of international ethical guidelines for biomedical research involving human subjects.

### Extraction of HBV DNA

HBV DNA was extracted from serum samples. Briefly, 100 μl serum was added to 400 μl buffer containing proteinase K (10 mM Tris-Cl pH 8.0, 10 mM EDTA pH8.0, 0.5% proteinase K) (Jingmei Biotech Co. Ltd.), water bath at 55°C for 3 hrs, and then DNA was extracted with chloroform and phenol.

### PCR amplification of HBV S gene

Modified primers for S gene were designed according to Yan et al. [19] and were synthesized by Beijing Auke Biotech Co. Ltd. Primer YS1, 5' ATGGGAATTCGGGGTTTTTCTTGTTGA 3'; Primer YS2, 5' CGTAAGCTTGGGACTCAAGATGTTGTA 3'. The product length is 586 bp. PCR was performed in 50 μl, 94°C denaturation for 4 minutes, and then 94°C for 50 seconds, 47°C for 1 min, 72°C for 1 min, totally 35 cycles were completed. The final extension was performed at 72°C for 5 min. The PCR products were directly sequenced and compared with AY167097.1, an epidemic HBV strain in China, by Fasta software.

## Abbreviations

HBV: Hepatitis B virus; HBV-GN: HBV-associated glomerulonephritis; MAPK: Mitogen-activated protein kinase; PTK: Protein tyrosine kinase; HBV-MN: HBV associated membranous nephropathy.

## Competing interests

The authors declare that they have no competing interests.

## Authors' contributions

LH and ZJ designed and carried out the study and wrote the manuscript. ZH carried out the serological and molecular biology assays. All authors read and approved the final manuscript.

## References

[B1] Pyrsopoulos NT Reddy KR Extrahepatic manifestations of chronic viral hepatitis Curr Gastroenterol Rep 2001 3 71 78 10.1007/s11894-001-0044-1 11177698

[B2] Sun L Xu H Zhou L Fang L Guo Y Effect of hepatitis B vaccine immunization on HBV associated nephritis in children Zhong hua Er Ke Za Zhi 2003 41 666 669 14733806

[B3] Han SH Extrahepatic manifestations of chronic hepatitis B Clin Liver Dis 2004 8 403 418 10.1016/j.cld.2004.02.003 15481347

[B4] Bhimma R Coovadia HM Hepatitis B virus-associated nephropathy Am J Nephrol 2004 24 198 211 10.1159/000077065 14988643

[B5] He XY Fang LJ Zhang YE Sheng FY Zhang XR Guo MY In situ hybridization of hepatitis B DNA in hepatitis B-associated glomerulonephritis Pediatr Nephrol 1998 12 117 120 10.1007/s004670050417 9543368

[B6] Ozdamar SO Gucer S Tinaztepe K Hepatitis-B virus associated nephropathies: a clinicopathological study in 14 children Pediatr Nephrol 2003 18 23 28 10.1007/s00467-002-0978-z 12488986

[B7] Ong HT Duraisamy G Kee Peng N Wen Siang T Seow HF Genotyping of hepatitis B virus in Malaysia based on the nucleotide sequence of pre S and S genes Microbes Infect 2005 7 494 500 10.1016/j.micinf.2004.12.016 15792534

[B8] Combes B Shorey J Barrera A Stastny P Eigenbrodt EH Hull AR Carter NW Glomerulonephritis with diposition of Australia antigen-antibody complexes in glomerular basement membrane Lancet 1971 2 234 237 4104770 10.1016/s0140-6736(71)92572-4

[B9] Han SHB Extrahepatic manifestations of chronic hepatitis B Clin Liver Dis 2004 8 403 418 10.1016/j.cld.2004.02.003 15481347

[B10] Willson RA Extrahepatic manifestations of chronic viral hepatitis Am J Gastroenterol 1997 92 3 17 8995930

[B11] Xu J Wang QX Fan CL Jiang D Li RB Cong X Fei R Chen HS Wei L Wang Y Comparison of hepatitis B virus serotype and genotype among HBsAg positive hepatitis B patients in a northern and a southern city of China Zhonghua Shi Yan He Lin Chuang Bing Du Xue Za Zhi 2003 17 327 329 15340543

[B12] Norder H Courouce AM Coursaget P Genetic diversity of hepatitis B virus strains derived worldwide: genotypes, subgenotypes, and HBsAg subtypes Intervirology 2004 47 289 309 10.1159/000080872 15564741

[B13] Iwasaki Y Tsuji T Serological subtype(serotype) of hepatitis B virus surface antigen Nippon Rinsho 1995 53 Suppl 293 298 12442399

[B14] Ohba K Mizokami M Ohno T Suzuki K Orito E Lau JY Ina Y Ikeo K Gojobori T Relationship between serotypes and genotypes of hepatitis B virus: genetic classification of HBV by use of surface genes Virus Res 1995 39 25 34 10.1016/S0168-1702(95)00069-0 8607280

[B15] Kim H Jee YM Song BC Shin JW Yang SH Mun HS Kim HJ Oh EJ Yoon JH Kim YJ Lee HS Hwang ES Cha CY Kook YH Kim BJ Molecular epidemiology of hepatitis B virus (HBV) genotypes and serotypes in patients with chronic HBV infection in Korea Intervirology 2007 51 52 57 10.1159/000096313 17164558

[B16] Kim SE Park YH Chung WY Study on hepatitis B virus pre-S/S gene mutations of renal tissues in children with hepatitis B virus-associated membranous nephropathy Pediatr nephrol 2006 21 1097 1103 10.1007/s00467-006-0168-5 16791604

[B17] Ma C Ji X Fang D Progress of hepatitis B virus S gene mutation Wei Sheng Wu Xue Mian Yi Xue Jin Zhan 2002 30 92 94

[B18] Levicnik-Stezinar S Hepatitis B surface antigen escape mutant in a first time blood donor potentially missed by a routine screening assay Clin Lab 2004 50 49 51 15000220

[B19] Yan L Hou J Guo Y Wang Z Lin Y Luo K Establish a new method of genotyping of hepatitis B virus by restriction pattern analysis of S ampicon Zhonghua chuanranbing zazhi 2001 19 224 228

